# Two or four injections of platelet-rich plasma for osteoarthritic knee did not change synovial biomarkers but similarly improved clinical outcomes

**DOI:** 10.1038/s41598-021-03081-6

**Published:** 2021-12-08

**Authors:** Srihatach Ngarmukos, Chotetawan Tanavalee, Chavarin Amarase, Suphattra Phakham, Warayapa Mingsiritham, Rangsima Reantragoon, Nitigorn Leearamwat, Thidarat Kongkaew, Kittipan Tharakhet, Sittisak Honsawek, Sinsuda Dechsupa, Aree Tanavalee

**Affiliations:** 1 Study Group of Biologics for Treatment of Knee Osteoarthritis, Chulalongkorn University, Bangkok, Thailand; 2 Department of Orthopaedics, Faculty of Medicine, Chulalongkorn University, 1873 Rama IV Road, Bangkok, 10330 Thailand; 3 Department of Orthopaedics, King Chulalongkorn Memorial Hospital, Bangkok, Thailand; 4 Department of Biochemistry, Faculty of Medicine, Chulalongkorn University, Bangkok, Thailand; 5 Immunology Division, Department of Microbiology, Faculty of Medicine, Chulalongkorn University, Bangkok, Thailand; 6 Department of Laboratory Medicine, Faculty of Medicine, Chulalongkorn University, Bangkok, Thailand

**Keywords:** Musculoskeletal system, Diseases, Outcomes research

## Abstract

We compared two and four intra-articular injections of platelet-rich plasma **(**PRP) in terms of changes of synovial cytokines and clinical outcomes. One hundred twenty-five patients having knee osteoarthritis (OA) underwent PRP injections at a 6-week interval. Before each PRP injection, synovial fluid aspiration was collected for investigation. Patients were divided into two or four intra-articular PRP injections (group A and B, respectively). Changes in synovial biomarkers were compared with the baseline levels of both groups, and clinical outcomes were evaluated until one year. Ninety-four patients who had completed synovial fluid collection were included for final evaluation, 51 in group A and 43 in group B. There were no differences in mean age, gender, body mass index (BMI), and radiographic OA grading. The average platelet count and white blood cell count in PRP were 430,000/µL and 200/ µL, respectively. There were no changes of synovial inflammatory cytokines (IL-1β, IL-6, IA-17A, and TNF-alpha), anti-inflammatory cytokines (IL-4, IL-10, IL-13, and IL-1RA), and growth factors (TGF-B1, VEGF, PDGF-AA, and PDGF-BB) between baseline levels and six weeks in group A, and 18 weeks in group B. Both groups had significantly improved clinical outcomes from six weeks including visual analog scale (VAS), patient-reported outcome measures [PROMs; Western Ontario and McMaster Universities Osteoarthritis (WOMAC) Index and Short Form-12 (SF-12)], with a significant delayed improvement of performance-based measures [PBMs; time up and go (TUG), 5-time sit to stand test (5 × SST), and 3-min walk test (3-min WT)]. In conclusion, two- or four-PRP intra-articular injection at a 6-week interval for knee OA demonstrated no changes of synovial cytokines and growth factors but similarly improved clinical outcomes from 6 weeks until 1 year.

## Introduction

Knee osteoarthritis (OA) is a significant degenerative joint disease affecting patients' quality of life and daily functions^1^. Treatment options are based on the stage of the disease, the patient's condition, and available medical facilities. Therefore, recommended methods range from non-surgical to surgical treatments^2–6^. However, in mild to moderate knee OA, non-surgical treatment options are in good agreement between physicians and patients with a wide diversity of results reported in the literature^2–4,6^.

The platelet-rich plasma (PRP), a biologic product prepared from autologous blood, has become an attractive option for the non-surgical treatment of knee OA^7–12^. However, there are variations of PRP in current use, including volume of collected blood^8^, number centrifugation of PRP preparation^9,10^, platelet activation before injection^12^, number of injection, and interval between each injection^7^. Studies regarding PRP applied in knee OA showed that it improved patient's clinical outcomes by releasing several growth factors and cytokines, promoting reparation and minimization of the inflammation occurring in the process of degenerative arthritis^7,8,12–17^. Some studies reported that PRP injection for knee OA provided better pain relief and improved functional outcomes than placebo control, hyaluronic acid (HA), or steroid injection^18–20^. However, major guidelines do not include or recommend against the use of PRP for the treatment of knee OA^3,5^.

The leukocyte-poor PRP (LP-PRP), a plasma preparation that has a high number of platelet cells with a low number of white blood cells, is an optional PRP for the treatment of knee OA to avoid mediating pro-inflammatory effect of white blood cells after the injection in knee OA^13,21,22^. The preparation of LP-PRP varies in terms of the number of centrifugation, the speed of centrifugation, and the type of collection tube. According to Filardo et al.^13^, using a commercial PRP kit, the single centrifugation technique was performed to separate the red blood cell from the other parts of the plasma. The supernatant PRP was transferred from the larger outer syringe into the small inner syringe and ready for use. Perez et al.^23^ proposed the double centrifugation technique, including the first centrifugation to separate the erythrocyte layer and the second centrifugation after collecting the upper plasma layer without the leukocyte layer (buffy coat). After the second centrifugation, the upper two/third volume of centrifuged plasma was removed, and the lower one/third was collected and defined as the LP-PRP for injection. Simental-Mendía et al.^22^ proposed similar steps of double centrifugation technique for LP-PRP; however, they used a faster speed of both centrifugations. Due to the heterogeneity of PRP preparation and protocol in knee OA treatment, a consensus of the French-spoken physicians has been carried out^24^. According to statements considered appropriate in this consensus, the characteristics of the injected PRP may influence the result in knee OA, the LP-PRP should be preferred, and a 4-to 8-mL volume of PRP with 1–3 injections could provide better outcomes^24^. Currently, a novel PRP classification has been proposed using the six-digit code (N1N2-N3N4-N5N6)^25^. The N1N2 represents platelet concentration in the drawn blood and in the PRP, the N3N4 represents red blood cells and white blood cells in PRP, and the N5N6 represents the external activation and calcium addition.

The present study compared the changes of inflammatory, anti-inflammatory cytokines, and growth factors in the synovial fluid between 2-cycle injection and 4-cycle injection of activated LP-PRP at a 6-week interval in knee OA patients, as well as patient's clinical outcomes, including pain relief using the visual analog scale (VAS), patient-reported outcome measures (PROMs), and performance-based measures (PBMs).

## Methods

This study was approved by The Institutional Reviewed Board (IRB) of The Faculty of Medicine, Chulalongkorn University, Bangkok, Thailand (COA No. 804/2020 and IRB No. 040/63). This study was registered at the Thai Clinical Trials Registry (TCTR), an identification number was TCTR20211108002; Clinical Trials Registry date 05/11/2021. According to the negative supporting of PRP treatment in major guidelines for knee OA treatment^3–5^, the approval from the IRB was limited to a prospective cohort study in patients who were well acknowledged and voluntarily chose one of two treatment protocols. Therefore, this study was neither controlled nor randomized of the two groups. All methods and experimental protocols were carried out in accordance with relevant guidelines and regulations. Informed consent was obtained from all patients who participated in the study.

From January to May 2020, all out-patients who had knee OA and came to the orthopedic clinic were introduced to a biologic treatment of knee OA with intra-articular injection of PRP. Selection criteria included knee OA according to the American College of Rheumatology (ACR)^26^, 45–85 years of age, grade 1 to 4 of radiographic knee OA according to the Kellgren-Lawrence system^27^, body mass index (BMI) < 30, VAS pain score > 4 at knee motion, > 90-degree arc of motion, < 10-degree anatomical knee deformity in the coronal plane, and no skin lesion around the affected knee. Exclusion criteria included inflammatory arthritis, previous knee surgery, knee pain with an antalgic gait, and thrombocytopenia. In addition, all recruited patients had a 2-week washing-out period for any pain killers or no non-steroidal anti-inflammatory drugs (NSAIDs), as well as joint supplements. Also, patients agreed not to take these medications during follow-up (FU). Among 140 eligible patients, 125 patients were enrolled in this study.

In the pilot study, the synovial fluid aspiration without an anesthetic agent was somewhat painful, especially in patients whose knees were not swollen. Therefore, patients were voluntary to choose 2-injection protocol (group A) or 4-injection protocol (group B). Patients were evaluated for clinical parameters at week 0 before the PRP injection and serial FU at 6 weeks, 12 weeks, 18 weeks, 6 months, and 1 year.

### PRP protocol and procedure

The LP-PRP was prepared by collecting a 30-mL of venous blood from the cubital vein with a blood collection tube containing anticoagulant citrate dextrose (ACD) solution (Vacuette, Greiner Bio-One, Austria). Another 5-mL peripheral blood was drawn for a complete blood count examination. The collected blood was centrifuged twice according to the technique described by Perez et al.^23^, whose in-vitro study reported a 70- to 80% platelet recovery and five-time higher platelet concentration than the peripheral blood with low white blood cell count. The first centrifugation was performed at 100G for 10 min to create three separate layers from the top to the bottom of the tube including, the plasma, the buffy coat, and the erythrocyte layers. The plasma layer was collected into a new sterile tube, and the second centrifugation was performed at 400G for 8 min. The upper two-third content of the plasma was then removed, and the remaining lower one-third plasma was then shaken to allow well mixing of the platelet cells and plasma. This portion was defined as the LP-PRP. Then, 1-mL of LP-PRP was collected for the analysis, similar to the complete blood count. The rest of LP-PRP, approximately 5.5–7.0 mL, was added with 0.1% volume of CaCl_2_ to produce activated LP-PRP before the injection.

Under a sterile preparation, the intra-articular LP-PRP injection was performed via the superolateral direction of the knee. Before every injection of another LP-PRP at all FU visits, the synovial fluid aspiration for molecular analysis was performed using a 23-gauge needle and a 5-mL syringe without anesthetic infiltration. First, a 1- to 2-mL synovial fluid was aspirated. The aspirated syringe was then dislodged from the needle, and then the syringe containing LP-PRP was relodged, and the injection procedure was performed. All steps of aspiration and LP-PRP injection were the same in all patients and all FU visits. Patients whose knee aspirations resulted in negative joint fluid or blood contamination were excluded from the study. The aspirated synovial fluid before the first LP-PRP injection (week 0) was defined as the baseline. In contrast, the rest were described as parameters after each intervention according to the time of FU.

After the PRP injection, patients were allowed to continue their daily activities as usual. No pain killers or anti-inflammatory drugs (NSAIDs) were permitted for any reason; otherwise, patients would be dropped out from the study. Patients in both groups were appointed for the next injection six weeks after the previous injection. Therefore, the PRP injection was performed at weeks 0 and 6 in group A and week 0, week 6, week 12, and week 18 in group B.

### Molecular study

The synovial fluid sample from each patient was centrifuged at 3500 rpm for 10 min and then stored at −80 °C until all the synovial fluid samples of all patients at all FUs were collected. Analysis for the level of all biomarkers was performed using a commercial proteome cytokine array kit (Bio-Plex suspension array system, BioRad, Hercules, CA, USA) according to the manufacturer’s instructions and reported in pg/mL. The molecular study, including levels of four synovial inflammatory cytokines (IL-1β, IL-6, IA-17A, and TNF-alpha), four anti-inflammatory cytokines (IL-4, IL-10, IL-13, and IL-1RA), and four growth factors (TGF-B1, VEGF, PDGF-AA, and PDGF-BB) of each patient were evaluated. All biomarkers of synovial fluid of patients in group A were compared between baseline values (week 0 before the first PRP injection) and those of week six before the second PRP injection. Similarly, the baseline levels of all biomarkers of patients in group B were compared to those of week 6, week 12, and week 18, obtained before the second, the third, and the fourth PRP injections.

### Clinical outcomes

**Figure 1:**
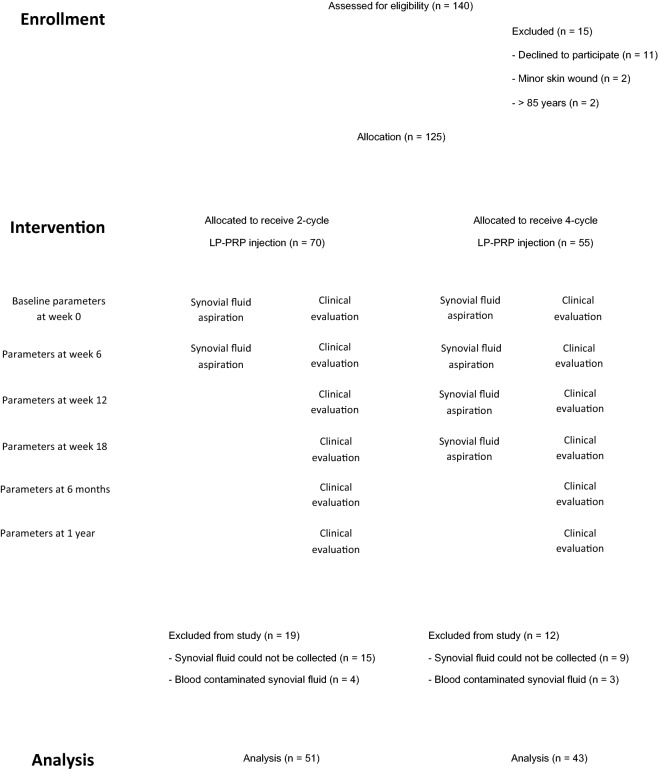
Demonstrating the study flow chart, which n represents the number of patients.

All patients were evaluated for clinical outcomes and followed until one year. At week 0 before the first PRP injection, all patients were evaluated for subjective pain using a visual analog scale (VAS) and patient-reported outcome measures (PROMs) using Western Ontario and McMaster Universities Osteoarthritis (WOMAC) index and Short Form-12 (SF-12). Another three tests of performance-based measures (PBMs), including time up and go (TUG), 5-time sit to stand test (5 × SST), and 3-min walk test (3-min WT), were evaluated by two of the authors using a digital stopwatch. All clinical outcome parameters were determined as baseline parameters. During the FU at six weeks, 12 weeks, 18 weeks, six months, and one year, all patients were again evaluated for VAS, PROMs, and PBMs. These parameters were assessed and compared to baseline values. The flow chart of the study is shown in Fig. 1.

### Sample size calculation and statistical analysis

The GraphPad Prism version 9.0.2, GraphPad Software, San Diego, CA, USA, https://www.graphpad.com, was used for statistical analyses. The sample size calculation was made according to the biomarker study of Kardos et al.^28^. We used the repeated one-way analysis of variance (ANOVA) and Tukey's posthoc test for sample size calculation. The effect size was 0.4. The beta error and the alpha error were 80% and 0.05, respectively. The total sample size was 80 (40 in each group). According to the technical problems related to the synovial fluid aspiration procedure (unable to collect synovial fluid or blood contamination in the synovial fluid), another 10% was added in each group to compensate for the drop-out. Therefore, the minimum allocated sample size was 44 in each group. The quantitative data were presented as mean and standard deviation. The independent t-test was performed to evaluate parameters among each group's baseline and other FU visits. The repeated one-way ANOVA followed by Tukey's posthoc test was performed to assess differences between groups A (2-cycle injection) and B (4-cycle injection).

### Ethical approval

This study was performed in line with the principles of the Declaration of Helsinki. Approval was granted by the Ethics Committee of Chulalongkorn University No IRB040/63. This study was registered at the Thai Clinical Trials Registry (TCTR), and identification number was TCTR20211108002; Clinical Trials Registry date 05/11/2021.

### Consent to participate

Informed consent was obtained from all individual participants included in the study.

### Consent to publish

Patients signed informed consent regarding publishing their data.

## Results

Among 125 patients in this studied group, 19 from 70 patients in group A and 12 from 55 patients in group B were excluded due to the inability to collect synovial fluid before PRP injection at any time or blood contamination in synovial fluid. Therefore, 51 patients in group A and 43 patients in group B were available for the evaluation. The patients' demographic data and radiographic grading of knee OA according to Kellgren-Lawrence (KL), ranging from KL 1, KL 2, KL 3, and KL 4, were shown in Table 1. Female patients were dominant, with an average age of 67 years. There were no differences between groups A and B in terms of mean age, gender, and BMI. The average PRP volume before injection was 6.7 mL (range, 5.5 to 7.2 mL) with 66.7% platelet recovery (range, 60 to 73%). The average platelet count in PRP was 430,000/µL (range, 350,000 to 610,000/µL) which was equal to 3.8 times of whole blood level, and the average white and red blood cell counts were 200/µL (range, 100 to 350/µL), 30,000/ µL (range, 20,000 to 45,000/µL), respectively. Therefore, according to Kon et al.^25^ regarding the classification and coding system, the PRP code in the present study was 24-00-11.

**Table 1:** Demographic data and comparison between group A (2 PRP injections) and group B (4 PRP injections) with no significant differences in all parameters.

Parameters	Studied group			*p*-value
	Overall	Group A	Group B	
		(2 PRP injections)	(4 PRP injections)	
No of patient	94	51	43	
Mean age (year)	67	67	66	0.79
No of male	7	4	3	0.59
No of female	87	47	40	
Mean BMI (kg/m^2^)	26.1	25.8	26.4	0.87
**Severity of OA**				
KL 1	2	1	1	0.35
KL 2	19	10	9	
KL 3	35	19	16	
KL 4	38	21	17	
Coronal deformiy (degree)	3.54º of AA	3.5º of AA	3.6º of AA	0.81
Range of motion (degree)	0–131	0–132	0–130	1

*KL* Kellgren and Lawrence, *AA* anatomical varus.

**Figure 2:**
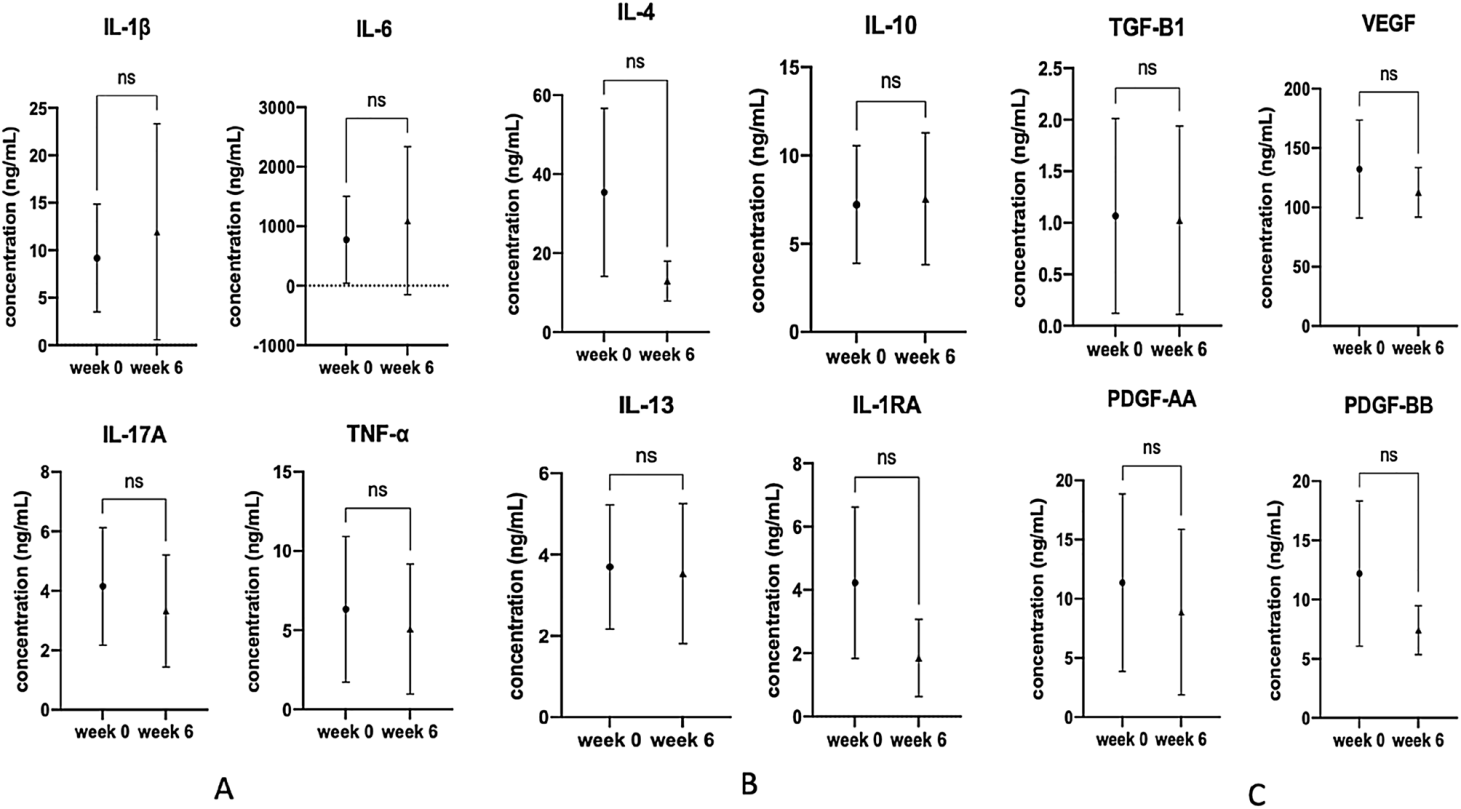
Demonstrating no differences in levels of synovial cytokines and growth factors of group A at six weeks of FU. The baseline levels were performed at week 0 before the first PRP injection. **(A)** Inflammatory cytokines including IL-1β, IL-6, IA-17A, and TNF-alpha. **(B)** Anti-inflammatory cytokines including IL-4, IL-10, IL-13, and IL-1RA. **(C)** Growth factors including TGF-B1, VEGF, PDGF-AA, and PDGF-BB.

**Figure 3:**
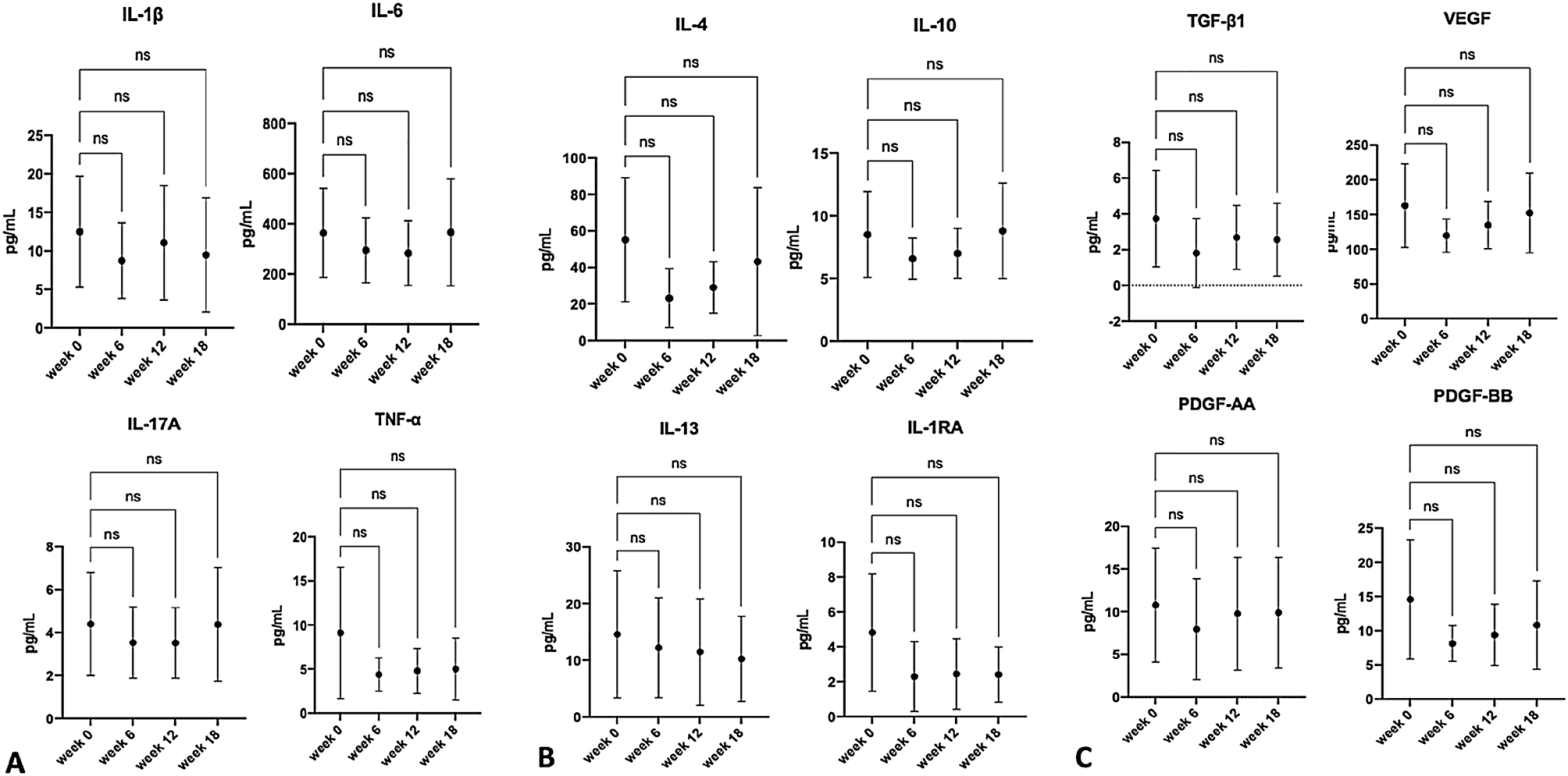
Demonstrating no differences in levels of synovial cytokines and growth factors of group B at 6 weeks, 12 weeks, and 18 weeks of FU. The baseline levels were performed at week 0 before the first PRP injection. **(A)** Inflammatory cytokines including IL-1β, IL-6, IA-17A, and TNF-alpha. **(B)** Anti-inflammatory cytokines including IL-4, IL-10, IL-13, and IL-1RA. **(C)** Growth factors including TGF-B1, VEGF, PDGF-AA, and PDGF-BB.

Regarding the molecular study of synovial fluid in both groups, all investigated parameters in group A were compared between week 0 and week 6, which were defined as parameters after one PRP injection. All investigated parameters in group B were evaluated from baseline (week 0), week 6, week 12, and week 18, defined as parameters after one PRP injection, two PRP injections, and three PRP injections. There were no significant changes in inflammatory cytokines (IL-1β, IL-6, IA-17A, and TNF-alpha), anti-inflammatory cytokines (IL-4, IL-10, IL-13, and IL-1RA), and growth factors (TGF-B1, VEGF, PDGF-AA, and PDGF-BB) in group A at six weeks compared to those at baseline as shown in Fig. 2. Similarly, there were no changes in the same investigated cytokines and growth factors at 6 weeks, 12 weeks, and 18 weeks compared to those at baseline, as shown in Fig. 3.

**Figure 4:**
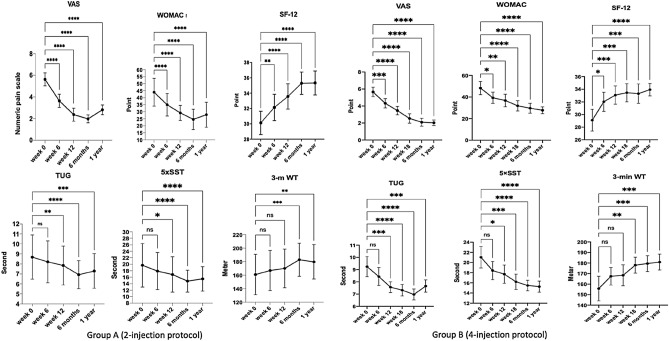
Demonstrating similar clinical outcomes of group A and B changes from baseline (week 0) to 6 weeks, 12 weeks, 18 weeks (only in group B), 6 months, and 1 year of FU. The VAS, WOMAC index, and SF-12 significantly improved from 6 weeks to one year in both groups. The TUG and 5 × SST significantly improved from 12 weeks to 1 year in both groups. The 3-m WT significantly improved from 6 months and 18 weeks to 1 year in group A and B, respectively (asterisks: *P ≤ 0.05, **P ≤ 0.01, ***P ≤ 0.001, and ****P ≤ 0.0001 vs baseline values)*.*

**Figure 5:**
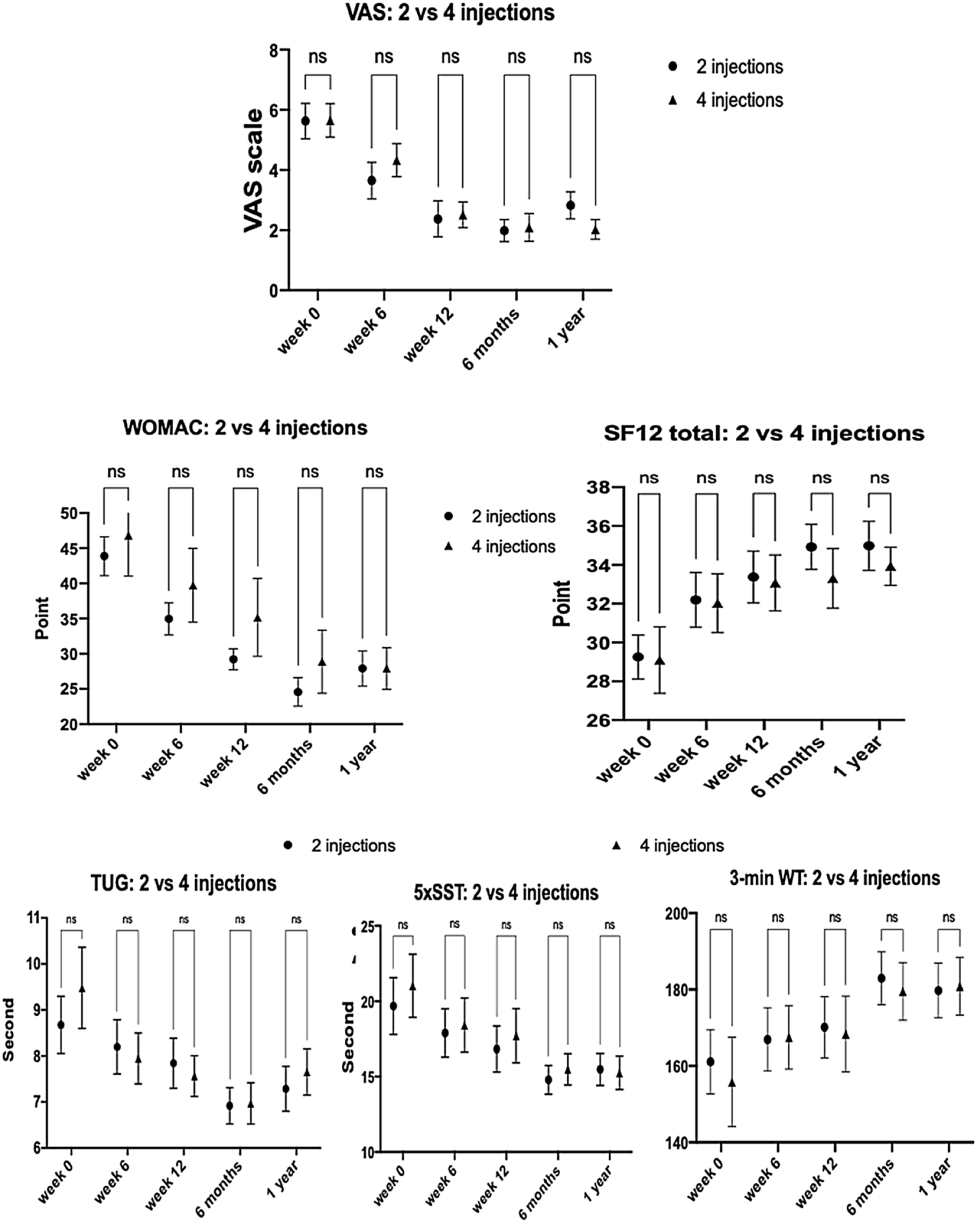
Comparing changes of clinical outcomes, including VAS, WOMAC, SF-12, TUG, 5 × SST, and 3-m WT between group A and B from the baseline (week 0) to 6 weeks, 12 weeks, 6 months, and 1 year after PRP injections with no significant differences between both groups at all FU visits.

Both groups A and B had a similar pattern of improved clinical outcomes. The VAS and the PROMs, including the WOMAC index and SF-12, significantly improved at 6 weeks after the baseline evaluation. However, the PBMs, including TUG, and 5 × SST showed significant improvement from 12 weeks, while the 3-min WT showed the latest progress from 18 weeks (Fig. 4). At 1 year FU, all parameters of clinical outcomes remained significantly improved from the baseline evaluation. Comparing clinical results between both groups A and B, all investigated parameters had similar significant improvement (Fig. 5).

## Discussion

The present study was designed to evaluate changes in inflammatory cytokines (IL-1β, IL-6, IA-17A, and TNF-alpha), anti-inflammatory cytokines (IL-4, IL-10, IL-13, and IL-1RA), and growth factors (TGF-B1, VEGF, PDGF-AA, and PDGF-BB) in synovial fluid and clinical outcomes related to two different protocols of LP-PRP injection at a 6-week interval. Patients in group A (2-injection protocol) were compared to group B (4-injection protocol) and were followed until 1 year. The results demonstrated three findings. Firstly, there were no changes in synovial inflammatory cytokines, anti-inflammatory cytokines, and growth factors in group A evaluated at 6 weeks before the second injection of PRP. Secondly, there were no changes in synovial inflammatory cytokines, anti-inflammatory cytokines, and growth factors in group B evaluated at 18 weeks before the fourth injection of PRP. Lastly, there was early significant improvement of clinical outcomes from 6 weeks, especially VAS and PROMs (WOMAC index and SF-12), and slower improvement of PBMs (TUG, 5 × SST, and 3-min WT) from 12 weeks, with no differences between both groups at all visits of FU until 1 year.

Studies have shown that the level of inflammatory cytokines in synovial fluid correlated with the severity of osteoarthritis^29–32^. Thus, clinical symptoms of OA should directly relate to the level of these cytokines. The present study used the LP-PRP for intra-articular injection according to the meta-analysis of Riboh et al., who reported that both LP-PRP and leukocyte-rich PRP (LR-PRP) provided no difference in efficacy or adverse events in knee OA treatment, but only results of LP-PRP had statistically superior to placebo and HA^33^. Although the meta-analysis of Meheux et al.^34^ recommended at least 2-cycle injections of PRP for knee OA, it has been unclear whether more injection cycles would provide better outcomes. So, we designed the present study to compare outcomes of 2-cycle (group A) vs. 4-cycle (group B) PRP injections, which created a 2-time difference in the number of injections. We evaluated levels of four synovial inflammatory cytokines, including IL-1β, IL-6, IA-17A, and TNF-alpha, four synovial anti-inflammatory cytokines, including IL-4, IL-10, IL-13, and IL-1RA, and four synovial growth factors, including TGF-B1, VEGF, PDGF-AA, and PDGF-BB and their changes from the baseline levels after multiple PRP injections of two different protocols, which would objectively reflect the effectiveness of PRP treatment. However, both groups had no significant synovial cytokines and growth factors changes at week six and week 18.

In contrast to the molecular study, all patients have significantly improved clinical outcomes from 6 weeks until 1 year. So, we found that there was no relationship between levels of synovial cytokines and clinical outcomes, as well as the number of PRP injections. Although the method of molecular research in the present study was a standard method in the biochemistry and immunology laboratories, the changed level cytokines and growth factors in synovial fluid might be too minimal to be detected. Therefore, a molecular study using other joint tissues, such as the synovial membrane, may be more appropriate.

In the present study, similarly improved VAS, WOMAC index, and SF-12 after PRP treatment agreed with several previous studies which reported significant pain relief and improved WOMAC index at one year of FU^9–11,18,35,36^. A recent meta-analysis including 34 randomized controlled trials in 1403 knees concluded that PRP provided better WOMAC than placebo and HA injection and better VAS than corticosteroid injection^37^. Besides the VAS and PROMs, the PBMs including TUG, 5 × SST, and 3-min WT were added in the present study to evaluate objective parameters of knee functions. These PBMs reflected a patient's actual function without any bias related to the patient's attitude on the PRP treatment. Although these three tests significantly improved, it took longer than the VAS and PROMs to gain a significant value. The 3-min WT was found delayed significant change compared to TUG and 5 × SST. The mean time to do TUG and 5 × SST in each FU visit took a much shorter time and less energy than 3-min WT. Therefore, more time and energy-consuming in the 3-min WT could be the primary cause to explain its delayed change among PBMs. According to differences of substantial time of improvement among investigated parameters, it is the straight foreword that a patient should have significant improvement of pain and PROMs before gaining improved PBMs, which relates directly to the consuming time, including TUG, and 3-min WT, respectively.

According to the PRP preparation protocol of the present study, we drew 30-mL peripheral blood with double centrifugation, which resulted in a mean of 6.7 mL of PRP. It contained a 3.8-time higher platelet cell count with a low leukocyte count. This PRP property was partially similar to that recommended in the French-speaking experts' current consensus statement on PRP treatment of knee OA^24^. This consensus suggested that the PRP should be prepared with double centrifugation, resulting in 4–8 mL, low leukocyte count, and 1- to 3-injection protocol several weeks apart. There were two different points of our study from their recommendation of the consensus. Firstly, the PRP in the present study has a lower platelet count than the recommended at 5-time or more concentration. Secondly, the present study included severe OA (KL 4) in both groups, which was not recommended by this consensus. However, in the severe OA subgroup, our selection criteria included only mild angular deformity resulting in minimal symptoms of mechanical knee pain that might have caused patients to undergo surgery^38^. The present study resulted in a favorable clinical outcome, despite lower platelet concentration in PRP and no change of molecular study. We believe that there are several factors affecting the positive effects of this biological treatment; however, the analysis of 12 synovial biomarkers in the present study did not provide any supportive evidence, so further studies to analyze the relationship between subjective findings related to patient’s clinical outcomes and objective results related levels of biomarkers may be helpful.

The weakness of the present study includes a non-randomized trial and no control group. We had to limit the study design to a prospective cohort study due to a lack of supporting guidelines on PRP treatment in knee OA, causing concern from the IRB. Also, the protocol, which performs repetitive knee aspirations for synovial fluid and injections of saline solution in the control group, did not get approval from the IRB. Therefore, we had to modify the protocol to prospectively compare baseline parameters (synovial biomarkers and clinical outcomes) with those evaluated at all visits of FU. Although both groups of patients voluntarily chose one of two protocols, there were no significant differences in all demographic data. This implied that both groups were indifferent. Therefore, both groups' results had similar unchanged molecular studies, and improved clinical outcomes could support that two or four LP-PRP injections for knee OA provided similar results.

Nevertheless, there are some strengths of the present study. Firstly, we evaluated synovial inflammatory cytokines, anti-inflammatory cytokines, and growth factors after one and three PRP injections which were invasive and technical demanding at the time of synovial fluid aspiration, resulting in indifference. Secondly, we demonstrated positive results of PRP with two different multiple injection protocols, which few studies focused on in this issue.

## Conclusions

Two- or four-cycle intra-articular injection of PRP for treatment of knee OA demonstrated similarly no significant changes in synovial inflammatory cytokines, anti-inflammatory cytokines, and growth factors at 6 weeks and 18 weeks after the first PRP injection, respectively. However, significantly improved VAS, PROMs (WOMAC and SF-12) could be demonstrated from 6 weeks until 1 year of follow-up with a significantly delayed improvement of PBMs (TUG, 5 × SST, and 3-min WT).

## Data Availability

The datasets generated in the current study are not publicly available because the PRP injection for knee osteoarthritis is not approved as a standard treatment in this country but are available from the corresponding author.
